# Therapeutic Potential of Glucagon-like Peptide-1 Receptor Agonists in Respiratory Disorders

**DOI:** 10.3390/ijms27135803

**Published:** 2026-06-26

**Authors:** Ewelina Russjan, Dominika Zając, Katarzyna Kaczyńska

**Affiliations:** Department of Respiration Physiology, Mossakowski Medical Research Institute, Polish Academy of Sciences, Pawińskiego 5, 02-106 Warsaw, Poland; erussjan@imdik.pan.pl (E.R.); dzajac@imdik.pan.pl (D.Z.)

**Keywords:** GLP-1, respiratory disorders, ALI/ARDS, asthma, COVID-19, pulmonary fibrosis, COPD, OSA

## Abstract

Glucagon-like peptide-1 (GLP-1) is an incretin hormone secreted in response to food intake that acts biologically by binding to GLP-1 receptors. The primary function of GLP-1 is to stimulate insulin secretion and inhibit glucagon secretion, which helps limit after-meal spikes in blood glucose. GLP-1 reduces intestinal contractility, slows down gastrointestinal motility and emptying, and also acts directly on the hypothalamus, thereby regulating appetite and food intake. Due to its metabolic effects, GLP-1 forms the basis of medications currently used to treat type 2 diabetes (T2DM) and obesity. However, it has also been observed that the use of GLP-1 agonists in the treatment of obesity or diabetes has a beneficial effect on comorbid respiratory conditions. This narrative review analyzes the scientific literature and describes the most recent information on the impact of GLP-1 receptor agonist (GLP-1 RA) therapies on the most common respiratory disorders—both the beneficial and undesirable effects. We discuss evidence that acute lung injury, COVID-19, pulmonary fibrosis, asthma, chronic obstructive pulmonary disease (COPD), and obstructive sleep apnea can benefit from therapies with various GLP-1 RAs. They can complement existing lung-targeted treatments, but as research progresses, they are likely to play an ever more important role in the treatment of respiratory diseases.

## 1. Introduction

Glucagon-like peptide-1 (GLP-1) is an incretin hormone produced in the intestinal epithelial endocrine L cells by differential processing of proglucagon, which is secreted in response to food intake. This hormone exerts its biological effects by binding to GLP-1 receptors, which are G protein–coupled transmembrane receptors (GPCRs), thereby increasing adenylate cyclase activity and cAMP levels. The main action of GLP-1 is to stimulate insulin secretion and to inhibit glucagon secretion by the pancreas, which helps to reduce postprandial glucose spikes. GLP-1 reduces intestinal contractility and slows gastric motility and gastric emptying, thereby acting as a physiological regulator of appetite and food intake. Additionally, the increased feeling of satiety is also due to the direct effect of GLP-1 on the hypothalamus [1,2,3]. Consequently, GLP-1, given its metabolic effects, has formed the basis for drugs currently used to treat type 2 diabetes (T2DM) and obesity [4]. Nevertheless, the effects of GLP-1 extend beyond traditional indications. In the pancreas, GLP-1 stimulates the proliferation of pancreatic β cells and prolongs their survival; in the kidneys, it induces mild natriuresis; in the cardiovascular system, it increases myocardial contractility and heart rate and improves vascular health; and in the brain, it shows antidepressant and neuroprotective actions [5,6].

It has also been noted that the use of GLP-1 receptor agonists (GLP-1 RAs) in the treatment of obesity or diabetes has a beneficial effect on coexisting respiratory conditions. Meta-analyses of the results of large randomized clinical trials (RCT), involving 55,922 and 77,485 participants using GLP-1 RAs, have shown a trend towards a reduced risk of bronchitis, pneumonia, upper respiratory tract infections, dyspnea, sleep apnea, acute respiratory failure, asthma, chronic obstructive pulmonary disease (COPD), pulmonary fibrosis, and pulmonary oedema compared with placebo [7,8]. A statistically significant association was also observed between the administration of GLP-1 RAs and the incidence of pulmonary oedema and bronchitis [8].

These effects may have been indirect, resulting from weight loss, improved lung compliance and capacity, and reduced external constriction; however, they may also have been due to the action on GLP-1 receptors, which are abundant in lung tissue—including immune cells, submucosal glands of the trachea, airway smooth muscle cells, lung epithelial and endothelial cells, and smooth muscle of the pulmonary arteries—where activation of the GLP-1R signaling pathway may cause increased mucus secretion, surfactant secretion, and relaxation of the respiratory muscles [9,10,11,12,13,14,15]. GLP-1 has been shown to modulate the inflammatory response by directly interacting with immune cells, adipocytes, and endothelial cells, thereby inhibiting nuclear factor-κB (NF-κB) activation—reducing the expression of pro-inflammatory cytokines and improving the inflammatory profile at both tissue and systemic levels [16,17,18,19]. Consequently, its agonists could become potential therapeutics for respiratory diseases in which inflammation plays a key pathogenic role, such as asthma, COPD, or lung inflammation [20]. Additionally, GLP-1 has protective effects against oxidative stress, which hastens the progression of many chronic lung conditions—such as COPD, asthma, and pulmonary fibrosis—by escalating inflammation, damaging cellular structures, and impairing lung function [21]. Numerous in vitro and in vivo studies have shown that GLP-1 and its receptor agonists reduce levels of reactive oxygen species (ROS) and protect against oxidative stress induced by increasing the expression of antioxidant enzymes and activating nuclear factor erythroid 2–related factor 2 (Nrf2) [22,23,24].

The aim of this narrative review is to analyze and discuss the scientific literature on the therapeutic potential of GLP-1 RAs, including their beneficial effects and side effects in respiratory diseases, based on data from both preclinical and clinical studies, as well as to attempt to explain the underlying mechanisms. This will be an update to the information, as research on the beneficial effects of GLP-1 RAs continues to grow each year, taking into account the latest scientific publications and disorders not covered in the previous comprehensive review [20], such as COVID-19, ALI/ARDS, and pulmonary fibrosis.

## 2. GLP-1R Agonists

GLP-1 receptor agonists (GLP-1 analogs) are a group of active substances used to treat type 2 diabetes and obesity with high efficacy. GLP-1 RAs are characterized by structural modifications, involving, in particular, changes to peptide bonds and amino acid substitutions, designed to prevent their breakdown by the enzyme dipeptidyl peptidase-4 (DPP-4) and to prolong their half-life. It is worth noting that natural GLP-1 has a very short half-life in plasma of 1.5 to 5 min [25]. GLP-1 RAs fall into two categories: human GLP-1-based drugs and exendine-4–based drugs. Drugs based on human GLP-1, modified to protect them from degradation by DPP-4, include dulaglutide, albiglutide (withdrawn from the market), liraglutide, and semaglutide. Exendin-4 is a natural peptide consisting of 39 amino acids, derived from the venom of the *Heloderma* lizards, which shares 53% similarity with human GLP-1. Drugs based on the structure of exendin-4 bind to and activate the GLP-1R with equal potency to native GLP-1. They are resistant to DPP-4 degradation and include exenatide, lixisenatide (withdrawn), and tirzepatide. The latter is a glucose-dependent insulinotropic polypeptide analog (GIP) and activates both GLP-1 and GIP receptors [3,26]. GIP is an incretin hormone secreted by K cells in the small intestine, mainly in reaction to glucose or fat intake, which enhances glucose-stimulated insulin release [12]. Therapy with GLP-1 RAs usually begins with a lower dose, which is then gradually increased over several weeks to improve tolerance of the medication and minimize side effects. Higher doses are typically related to greater weight loss and better glycemic control, but may also increase the risk of side effects. The mode and frequency of administration, as well as the dosage ranges for GLP-1 RAs, are shown in Table 1. They are generally safe, and their safety profile has been well established through many years of use, although they often cause gastrointestinal side effects such as nausea, vomiting, and diarrhea, which are usually dose-dependent and decline over time [27]. Among severe gastrointestinal disorders, pancreatitis is one of the most commonly reported adverse effects; however, there is a lack of statistically significant studies confirming this association [28]. Other adverse effects observed include renal problems, dizziness, mild tachycardia, infections, headaches, and indigestion [3,29]. A potential issue is the development of immunogenicity in some patients, who may produce antibodies against certain GLP-1 analogs, which could affect their efficacy and lead to injection-site reactions or even anaphylactic shock, as in the case of exenatide [3].

## 3. GLP-1 RAs and Acute Lung Injury (ALI)

Acute lung injury (ALI) is a form of acute pneumonia characterized by dysfunction of the pulmonary vascular endothelial barrier and the alveolar epithelial barrier. ALI may rapidly progress to acute respiratory distress syndrome (ARDS), characterized by widespread lung inflammation, uncontrolled oxidative stress, neutrophil accumulation, pulmonary oedema, severe hypoxemia, and respiratory failure, which is potentially directly life-threatening. The mortality rate can be as high as 43% [32]. ALI/ARDS can be induced by multiple factors, such as sepsis, bacterial or viral lung infection, trauma, harmful gas inhalation, or drug toxicity [33,34]. Management of patients with severe ARDS has become a major clinical challenge due to its rapid progression and life-threatening outcome. Treatment entails mechanical support of the patient’s breathing while the lungs heal, or, in severe cases, extracorporeal membrane oxygenation (ECMO) [35]. To date, there is no cure for ALI/ARDS, although recent attention to the use of GLP-1 RAs in experimental studies has demonstrated their potency [36,37]. Prior treatment with liraglutide reduced lipopolysaccharide (LPS)-/sepsis-induced ALI in mice, most possibly through its interaction with the TxNIP/NRLP3 inflammasome pathway, manifested by a decrease in lung injury and inflammation, a reduced number of immune cells and total protein level in bronchoalveolar lavage fluid (BALF), and a reduced expression of inflammatory cytokines in BALF and lung tissue [38,39,40]. Also, in the H9N2 influenza virus–induced ALI/ARDS mouse model, the efficacy of liraglutide in alleviating the severity of lung damage, reducing inflammatory cell infiltration and total protein levels in BALF, and reducing mortality was attributed to a decrease in the levels of pro-inflammatory cytokines (IL-1β, IL-6, and TNF-α) in BALF [41].

In addition to its anti-inflammatory effect, GLP-1 RAs regulate the expression and secretion of surfactant proteins, which can be vital for increasing lung compliance and reducing edema in ALI. This is evident in the study with *Pseudomonas aeruginosa*-induced sepsis and ALI, where mice treated with liraglutide showed not only reduced levels of inflammatory cells, pro-inflammatory cytokines (TNF-α and IL-6), and reduced mortality, but also increased expression and secretion of pulmonary surfactant proteins (SP-A and SP-B) and phospholipids [42]. Previous study has shown that liraglutide increased SP-A expression in lung alveolar epithelial type II cells and alleviated lung inflammation and edema in LPS-induced ALI, most likely via the thyroid transcription factor-1 (TTF-1) signaling pathway [43]. A study with another GLP-1 RA, dulaglutide, showed that inhibition of epithelial cell apoptosis may be a promising strategy for treating ALI. Administration of dulaglutide reduced lung damage, decreased expression of IL-1β, TNF-α, IL-6, CXCL1, CCL2, and CXCL2, and reduced neutrophil and macrophage infiltration in lung tissue. Simultaneously, the increase in the expression of caspase-3, cleaved caspase-3, caspase-8, and Bcl-2/Bax and the decrease in the number of TUNEL-positive cells in the lungs suggest that this GLP-1 RA may play a protective role in the lungs by inhibiting not only inflammation but also apoptosis [44].

Another aspect of GLP-1R activation is the maintenance of the functional endothelial barrier and inhibition of polymorphonuclear neutrophil (PMN) extravasation via inhibition of Rho/NF-κB signaling. In cultured human pulmonary microvascular endothelial cells, liraglutide prevented LPS-induced endothelial barrier damage by restoring intercellular tight junctions and adherens junctions. It also prevented PMN adhesion to the endothelium by inhibiting the expression of intercellular adhesion molecule-1 (ICAM-1) and vascular cell adhesion molecule-1 (VCAM-1) and thereby suppressed PMN migration through the endothelium [45]. The data described above regarding the multifaceted effects of GLP-1 RAs in ALI point to their significant potential; however, these studies have so far been conducted only in vitro and in animal models (Table 2). Further research is therefore required, including confirmatory clinical trials.

**Figure 1:**
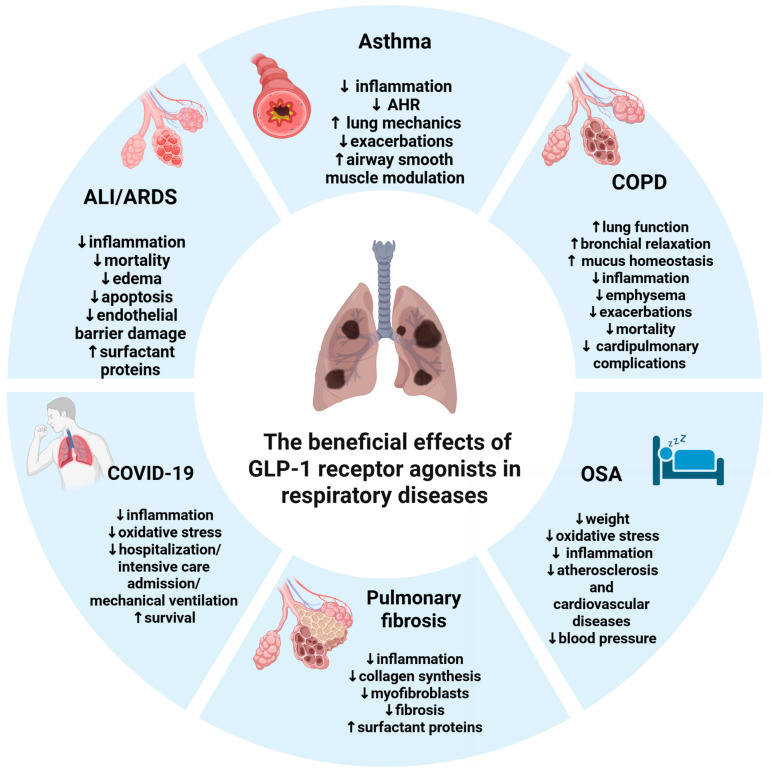
The beneficial effects of GLP-1 receptor agonists in respiratory disorders. AHR: airway hyperresponsiveness; ALI/ARDS: acute lung injury/acute respiratory distress syndrome; COPD: chronic obstructive pulmonary disease; OSA: obstructive sleep apnea; ↓: decrease; ↑: increase.

The benefits of GLP-1 RAs in relation to various respiratory diseases identified in this review, including ALI/ARDS, are summarized in Figure 1.

## 4. GLP-1 RAs and COVID-19

Infection with the SARS-CoV-2 coronavirus can take a severe form in the course of COVID-19, the 2019 coronavirus disease, resulting in pneumonia leading to ARDS [46]. Diabetes, particularly when uncontrolled, is recognized as a significant risk factor for morbidity and mortality due to COVID-19. As GLP-1 RAs are a group of medicines used primarily to treat type 2 diabetes, their use might mitigate these risks. Indeed, there are a few individual studies and meta-analyses concerning COVID-19 and diabetes patients showing that treatment with GLP-1 RAs was associated with lower odds of hospitalization [47,48], admission to intensive care and/or mechanical ventilation [48], and mortality compared with controls [48,49,50,51]. In addition, the beneficial effects of GLP-1 RAs appear to be sufficient to contribute to an increase in overall survival in people with diabetes, even over a two-year follow-up period [52]. Independent of their glucose-lowering actions, GLP-1 RAs could further support the treatment of COVID-19 through their anti-inflammatory and antioxidant effects [53].

It is recognized that GLP-1 reduces inflammation by inhibiting the NF-κB signaling pathway and related cytokine release, which, when excessive and uncontrolled, can induce the so-called cytokine storm—a key factor in severe COVID-19 cases [54,55,56,57]. Particularly pathologically elevated levels of pro-inflammatory cytokines, such as IL-1β, IL-6, and TNF-α, are responsible for deterioration in the patient’s condition and underlie key pathophysiological symptoms in patients with severe COVID-19 [58]. Notably, the anti-inflammatory effects of GLP-1 RAs have been evidenced in the clinical setting, where liraglutide therapy reduced serum levels of TNF-α, IL-6, IL-1β, and the inflammatory macrophage activation molecule sCD163 in patients with T2DM [59]. In subsequent studies on T2DM patients, the GLP-1 RAs liraglutide and semaglutide reduced elevated levels of IFN-γ, TNF-α, and IL-6 [60,61]. In addition, liraglutide was able to decrease blood IL-6 levels in patients with type 1 diabetes [62]. Exenatide, another GLP-1 RA, was administered over a 12-week period in T2DM patients with obesity and suppressed a number of inflammatory processes in blood mononuclear cells (MNCs), including the nuclear binding of NF-κB and the expression of TNF-α, JNK-1, TLR-2, TLR-4, IL-1β, and SOCS-3. Furthermore, a decrease in the plasma concentrations of MCP-1, serum amyloid A (SAA), IL-6, and matrix metalloproteinase-9 (MMP-9) was observed, and the anti-inflammatory effect was not dependent on weight loss, as no such reduction was observed [63]. Finally, human studies have also demonstrated the effect of GLP-1 RAs on reducing serum C-reactive protein (CRP) levels—a strong predictor of COVID-19 severity and mortality. Reduced hsCRP was shown after exenatide [64] and semaglutide [65], and reduced CRP was shown after semaglutide therapy [66].

Experimental evidence suggests a potential anti-inflammatory effect of GLP-1 RAs in a COVID-19 model, as liraglutide alleviated LPS-induced endotoxemia in rats by inhibiting pro-inflammatory mediators, such as IL-6, TNF-α, monocyte chemotactic protein-1 (MCP-1), VCAM-1, and ICAM-1 [67].

In addition to their anti-inflammatory effects, there are also studies indicating that GLP-1 RAs may exhibit significant antioxidant effects, which is relevant in the context of SARS-CoV-2 infection. For example, liraglutide reduced whole-blood and aortic reactive oxygen species (ROS) formation in LPS-induced endotoxemic rats [67], and exanatide reduced the production of ROS in the blood mononuclear cells of diabetic patients [63]. Further, treatment of human macrophages from healthy volunteers with exenatide in the presence of LPS resulted in a decrease in reactive oxygen species (ROS) and malondialdehyde (MDA) levels by reducing the expression of ROS-generating NADPH oxidase and by increasing the expression and activity of antioxidant enzymes superoxide dismutase (SOD) and glutathione peroxidase (GSH-Px) [68]. The information presented above indicates that GLP-1 RAs have broad therapeutic potential extending beyond their current use in glycemic control, owing to their effects on inflammatory processes and oxidative stress. It can be assumed that they may also offer therapeutic benefits in combating SARS-CoV-2 infection and COVID-19, particularly as the GLP-1R is widely expressed in the lungs, and its activation—leading to the inhibition of cytokine release and oxidative stress—could limit the development and progression of ARDS associated with severe COVID-19 [53,69]. Another area requiring further confirmatory clinical trials is the evaluation of the effects of GLP-1 RAs on patients with the so-called long COVID, defined as symptoms persisting for at least 3 months after recovering from acute COVID-19 [70]. In a promising retrospective cohort study of adult T2DM patients, which analyzed treatment outcomes over a 12-month period following acute COVID-19 with regard to GLP-1 RA use, a beneficial effect was observed on all-cause mortality, cognitive impairment, epilepsy/seizures, and thromboembolic diseases—conditions associated with long COVID [71].

## 5. GLP-1 RAs and Pulmonary Fibrosis

Pulmonary fibrosis (PF) is a progressive lung disease marked by scarring of lung tissue, impaired respiratory function, and impeded gas exchange. The known factors contributing to the development of pulmonary fibrosis include chronic inflammation, autoimmune diseases, environmental factors (dust, asbestos, and smoking), certain medications (cytostatics), radiotherapy, and infections (COVID-19, tuberculosis, fungal infections, and others). Permanent exposure to them causes repeated microdamage to alveolar epithelial cells, leading to dysregulated repair mechanisms based on myofibroblast proliferation and excessive amounts of extracellular matrix components, resulting in scarring and stiffening of lung tissue [72,73]. Molecular pathways mediating various aspects of cell proliferation, differentiation, apoptosis, and promotion of fibrosis progression include the TGF-β/Smad, WNT/β-catenin, and PI3K/Akt/mTOR signaling pathways [74].

PF is distinguished by high death rates, a poor life expectancy, and poor response to available medical therapies, i.e., treatment with glucocorticoids, immunosuppressants, and antifibrotic agents. The progression of fibrosis can be slowed down but not reversed, with a 5-year survival rate for patients with PF under 50% [74,75].

Due to the limited and ineffective treatment of PF, the search for new therapeutic approaches continues. In recent years, there has been growing interest in the role of GLP-1 and its receptor in alleviating pulmonary fibrosis. The evidence available so far focuses on experimental models of PF and in vitro studies, with promising results. In mice with bleomycin (BLM)—induced pulmonary fibrosis—that were given liraglutide at a dose of 2 mg/kg/day for 27 days, a reduction in pulmonary inflammation and fibrosis was observed, most likely through the inactivation of NF-κB [76]. In particular, liraglutide decreased lung overexpression of α-smooth muscle actin (α-SMA), VCAM-1, and TGF-β1; reduced the DNA binding activity of NF-κB p65; and limited the number of macrophages and lymphocytes in BALF. Likewise, in the rat BLM model, liraglutide administered at a significantly lower dose of 100 µg/12 h in two treatment modes, between day 1 and day 6 and between day 10 and day 20 after intratracheal administration of BLM, was highly effective at both the pro-inflammatory and fibrosis phases of the disease [77]. Activation of the GLP-1 receptor reduced the expression of collagen mRNA, hydroxyproline, a number of enzymes critical for collagen synthesis, the presence of myofibroblasts, and the expression of cytokines promoting fibrosis (Tgfb1 and Ctgf), thereby favoring partial restoration of the alveolar structure. The GLP-1 RA increased the production of surfactant proteins (SFTPa1, SFTPb, and SFTPc) and also regulated the activity of pulmonary angiotensins and their receptors, thereby improving the condition of the pulmonary vessels and heart function. One of the most recent studies showed that GLP-1 RA treatment inhibited fibroblast activation in vitro and alleviated silica-induced pulmonary inflammation and fibrosis in mice treated with liraglutide (150 μg/kg daily) for 56 days [78]. GLP-1R activation had an antifibrotic effect by interfering with the interaction between the NLRP3 inflammasome and PFKFB3-mediated glycolysis, followed by inhibition of histone lactylation in fibroblasts. This led to a downregulation of profibrotic gene expression in activated pulmonary fibroblasts. In another recent study, which used a formulation containing gelatin-coated chitosan microparticles to deliver semaglutide to the lungs in a BLM rat model, a significant reduction in inflammation was demonstrated via inhibition of the TLR4/NFκB signaling pathway and a reduction in pro-inflammatory cytokine levels. This slowed down fibrosis progression, as reflected by a reduction in the concentration of fibrosis mediators—including TGF-β1 and p-SMAD3—as well as markers such as α-SMA and Col1a1 in lung tissue [79]. In summary, the GLP-1R is a promising therapeutic target for the treatment of pulmonary fibrosis. However, the beneficial effects of GLP-1 RAs have so far been limited to experimental models and, therefore, need to be evaluated in clinical trials.

## 6. GLP-1 RAs and Asthma

Bronchial asthma is a chronic respiratory disease associated with characteristic symptoms such as shortness of breath, chest tightness, wheeze, and cough, which can change over time. It is typically correlated with airway inflammation and hyperresponsiveness [80]. Asthma, which affects over 300 million patients worldwide, is heterogeneous in nature, and several different clinical phenotypes of the disease are currently distinguished [81]. One of these is obesity-related asthma, as being overweight or obese significantly increases the risk of developing asthma, and many people with severe or difficult-to-treat asthma have obesity [80,82]. Excess body fat in the abdominal and chest areas plays a key role in the interaction between these two conditions, as it directly affects lung function. This can result in incorrect positioning of the diaphragm, which in turn reduces lung volume and increases the need for ventilation [83]. Equally important is the fact that adipose tissue is a metabolically active organ, and, in patients with obesity, produces increased levels of pro-inflammatory mediators, leading to low-grade chronic inflammation that further exacerbates existing inflammation in the airways [84]. It is assumed that in cases of asthma coexisting with obesity or metabolic syndrome, there is a decrease in GLP-1 levels, increased insulin resistance, as well as reduced nitric oxide production and increased smooth muscle contractility, which together may lead to bronchoconstriction [85]. Despite the growing number of asthmatics with obesity, there are no specific recommendations regarding treatment for this group of patients. However, considering the anti-inflammatory activity of GLP-1 [17] and the presence of GLP-1 receptors (GLP-1Rs) in the respiratory tract [86]—as well as the established role of GLP-1 RAs in the pharmacotherapy of type 2 diabetes mellitus or obesity [87]—a natural direction for research appears to be the use of these drugs in the treatment of asthma complicated by metabolic diseases.

Importantly, apart from an anti-inflammatory effect, GLP-1 RAs exhibit other mechanisms that may beneficially affect asthma control, such as reducing airway hyperresponsiveness (AHR), improving lung mechanics, or modulating airway smooth muscle cells [88], which is reflected in the results of human studies and in animal models of the disease. Zhu et al. demonstrated that in a mouse model of ovalbumin (OVA)-induced asthma, liraglutide reduced airway inflammation and mucus secretion, which was probably related to protein kinase A (PKA)-dependent inactivation of the NF-κB signaling pathway [89]. In another study using *Alternaria alternata* extract as an aeroallergen, administration of a GLP-1R agonist two days before the first challenge suppressed IL-33 release into the BALF and its expression in lung epithelial cells. The use of liraglutide reduced the number of group 2 innate lymphoid cells (ILC2s) expressing IL-5 and IL-13 and decreased lung eosinophilia, mucus production, and airway reactivity. Administration of liraglutide one day after the first *Alternaria* challenge also produced beneficial effects, including a reduced number of eosinophils and decreased lung expression of type 2 cytokines and chemokines [90]. Similarly, *Alternaria* extract was used to induce asthma symptoms in a polygenic mouse model of obesity (TALLYHO mice), in which the studied animals exhibit hyperinsulinemia, hyperglycemia, and hyperlipidemia [91]. Administration of a GLP-1 RA resulted in reduced release of IL-33 and thymic stromal lymphopoietin (TSLP) in BALF, decreased the number of ILC2 cells, decreased lung expression of IL-5 and IL-13, decreased ICAM-1 expression on lung endothelial and epithelial cells, and declined airway resistance to methacholine. Additionally, liraglutide reduced airway neutrophilia and the production of chemoattractants for neutrophils, such as KC and LIX. These results clearly indicate the possibility of using GLP-1 RAs in patients with obesity concurrently suffering from bronchial asthma [91]. Moreover, liraglutide demonstrated effectiveness in another model of obese asthmatic mice in which obesity was the result of a high-fat diet (HFD), and asthma was induced by OVA sensitization and challenge. Treatment with the GLP-1 RA led to significant weight loss, reduced eosinophilic airway inflammation, reduced expression of IL-4, IL-5, and IL-33 in BALF, as well as suppressed airway hyperresponsiveness and NLRP3 inflammasome activity in lung tissue [92]. Another GLP-1 RA that shows promising effects in alleviating asthma symptoms is dulaglutide. Its potential was studied in a mouse model of disease with the assumption that, if weight reaches or exceeds 145% of the initial value, obesity alone, without additional allergen sensitization, can lead to non-Th2, non-eosinophilic asthmatic inflammation. Dulaglutide attenuated HFD-induced symptoms such as weight gain, elevated levels of leptin and insulin in serum, an elevated number of macrophages in BALF, elevated levels of IL-17, IL-1β, and transforming growth factor (TGF)-β1 in lung homogenates, airway hyperresponsiveness, lung fibrosis, and enhanced differentiation of Th1 and Th17 cells. Interestingly, these positive effects were intensified with the co-administration of dulaglutide with another antidiabetic drug, empagliflozin [93]. Nevertheless, there is a study that calls into question the protective role of GLP-1 RAs in lung inflammation. In a mouse model of HFD-induced obesity with intranasal house dust mite challenge and bariatric surgery, GLP-1R deficiency had a limited effect on markers of allergic airway inflammation or on the gut–lung microbiota axis [94]. On the other hand, in obese mice with aeroallergen-induced asthma, anti-allergic activity in the airways was demonstrated for tirzepatide, a dual agonist of the GIPR and GLP-1R [95]. In subsequent studies using GLP-1R knock-out (KO), GIPR KO, and GLP-1R/GIPR double-KO mice, the involvement of individual endogenous signaling pathways in modulating the inflammatory state was assessed. In comparison to the other strains, animals with a deficiency of both incretin receptors were characterized by a higher concentration of TSLP in BALF, increased infiltration of lymphocytes, eosinophils, and neutrophils, an elevated number of ILC2 cells, enhanced expression of IL-5 and IL-13 in the lung, and enhanced expression of ICAM-1 in epithelial cells after sensitization with *Alternaria alternata* extract. These results suggest that simultaneous activation of both GLP-1 and GIP receptors, rather than stimulating each of them separately, may be a more effective way of alleviating asthmatic inflammation in the airways [96]. As previously mentioned, obesity-related asthma is currently considered a separate phenotype of the disease, and it is worth noting that in individuals with obesity, T2DM and insulin resistance are independent risk factors for asthma exacerbations and poor disease control [97]. Hence, there are several studies on the use of GLP-1 RAsin this group of patients. Khan et al. observed that in patients with diabetes and asthma, 52-week treatment with liraglutide resulted in weight loss and significantly improved asthma control, including a reduction in the number of disease exacerbations [98]. Moreover, in the randomized crossover LIRALUNG study, liraglutide showed a beneficial effect on forced vital capacity (FVC) in diabetic patients compared to placebo [99]. Another long-term observational study showed that the group of T2DM patients treated with GLP-1 RAs was characterized not only by increased FVC, but also by a significant increase in forced expiratory volume in 1 s (FEV_1_) and maximal expiratory flow at 50–75%, which indicates the potential use of these drugs in respiratory disorders [100]. Some studies, in turn, focus on comparing the potential of various antihyperglycemic drugs in alleviating asthma symptoms. Foer et al. have demonstrated that adult diabetic patients with asthma who were prescribed GLP-1 RAs exhibited milder symptoms and a lower number of asthma exacerbations compared to individuals using sodium-glucose cotransporter-2 (SGLT-2) inhibitors, DPP-4 inhibitors, sulfonylureas, or basal insulin for intensification of T2DM treatment [101]. Similarly, diabetics receiving GLP-1 RAs had fewer exacerbations of chronic lower respiratory diseases (asthma and/or COPD) compared to patients treated with DPP-4 inhibitors [102]. In another cohort study, it was also shown that metformin reduces the risk of asthma attacks, and adding a GLP-1 RA exerts a beneficial synergistic effect. This phenomenon was probably related to mechanisms other than glucose level control or weight loss [103].

Nevertheless, the results of subsequent studies are contradictory, which significantly complicates determining the actual influence of GLP-1 RAs on bronchial asthma.

Data from a meta-analysis of randomized placebo-controlled trials with cardiorenal outcomes indicate that SGLT-2 inhibitors may reduce the risk of asthma incidents, whereas DPP-4 inhibitors and GLP-1 RAs do not show a considerable impact on this rate [104]. This is confirmed by another meta-analysis encompassing 39 studies, which demonstrated a moderate reduction in the frequency of asthma occurrence in individuals with T2DM or obesity using GLP-1 therapy in comparison to non-users [105]. Another population-based study showed that the use of GLP-1 RAs and DPP-4 inhibitors was related to a greater risk of asthma exacerbations with the necessity of systemic corticosteroid administration [106]. Additionally, data from the Food and Drug Administration (FDA) adverse event reporting system revealed that specific GLP-1 RAs, such as liraglutide and semaglutide, may be associated with an increased number of asthma or asthma-like adverse events, and exenatide is characterized by the most frequent occurrence of serious adverse effects, including cases of death. The authors emphasize that the observed relationships may be primarily statistical in nature, rather than causal, and should be interpreted with caution [107].

The results of the latest studies, however, suggest a beneficial role of GLP-1 RAs in asthma since, in a retrospective cohort study, they reduced the risk of developing asthma by 33% in patients with T2DM, and the protective effect was present at different levels of asthma severity, except in patients who required endotracheal intubation [108]. In an observational real-world study involving asthmatics with obesity, GLP-1 RAs improved asthma control, but there was no difference in the asthma exacerbation rate compared to the unexposed group [109]. Further research suggests that the greatest benefits of GLP-1 RA treatment are experienced by patients with asthma in whom the severity of the disease has led to visits to the emergency department [110], as well as by those who have two or more prescriptions for oral steroids [111]. Another systematic review, considering several previously cited studies, indicated a reduced risk of asthma and COPD exacerbations related to the use of GLP-1 RAs in adults with obstructive lung diseases and T2DM compared to patients treated with sulfonylureas and DPP-4 inhibitors [112]. The latest meta-analysis from 2026, encompassing 27 large randomized controlled trials, compared the effects of novel glucose-lowering drugs on the risk of various respiratory diseases and showed that GLP-1 RAs decreased the risk of respiratory failure, pneumonia, and asthma in patients with obesity [113]. It is also worth highlighting that a randomized, placebo-controlled, double-blind clinical trial is currently being conducted to investigate the effect of semaglutide, one of the GLP-1R agonists, on asthma control and airway inflammation in adult asthmatics with obesity, which will certainly help clarify the role of GLP-1A in regulating asthma symptoms [114].

## 7. GLP-1 RAs and Chronic Obstructive Pulmonary Disease (COPD)

Chronic obstructive pulmonary disease is defined as a persistent limitation of airflow through the airways, which is usually progressive and occurs in association with an exaggerated chronic inflammatory response of the airways and lungs to noxious particles, gases, and, in most cases, tobacco smoke. Chronic inflammation causes structural changes in the airways, lung parenchyma (emphysema), and pulmonary vessels, resulting in restricted airflow and reduced lung capacity. The symptoms include coughing, shortness of breath, and sputum production. Bronchial obstruction and dyspnea are initially felt during physical exertion, but as the disease progresses, they also occur at rest, significantly impairing quality of life. Ultimately, the disease can lead to respiratory failure and death. Exacerbations and comorbidities add to the overall severity of the disease [115,116]. Obesity and metabolic syndrome often occur simultaneously with COPD, and their prevalence in COPD ranges from 21% to 57% [117,118]. The incidence of diabetes in COPD patients is also higher (25%) than in the general population, and patients with COPD and diabetes are at greater risk of severe exacerbations and death than COPD patients without diabetes [44]. Given that GLP-1 RAs are commonly used in patients with diabetes and obesity and exhibit hypoglycemic, anti-inflammatory, antioxidant, and vascular protective effects, and that the GLP-1 receptor is widespread in the lungs, it can be argued that GLP-1 and its agonists may play a therapeutic role in patients with COPD [119,120,121]. Interestingly, airway smooth muscle (ASM) cells and peripheral blood mononuclear cells (PBMCs) from patients with COPD show significantly reduced expression of the GLP-1 receptor (GLP-1R), suggesting that this deficiency could be modulated by GLP-1 RAs [122,123]. Notably, overexpression of the GLP-1R in isolated human airway smooth muscle (ASM) cells inhibited their proliferation, migration, and the cytokine release of IL-1β, IL-4, TNF-α, and GM-CSF. This effect occurred through an increase in the expression level of the adenosine triphosphate-binding cassette, subfamily A, member 1 (ABCA1) gene in bronchial smooth muscle cells, as it was abolished by silencing the ABCA1 gene using siRNA [122]. The data from experimental models of obstructive lung diseases do not always support the effects of GLP-1 RAs on pro-inflammatory cytokine production. In a mouse model in which exacerbations were induced by inhalation of ovalbumin and LPS, GLP-1 RAs (liraglutide or exenatide) improved the survival of mice and their lung function via the induction of bronchial relaxation, but had no effect on surfactant expression or inflammatory cytokine levels [124]. In another study conducted with the same model, an improvement in lung function was also observed, and it was suggested that the bronchodilatory effect of liraglutide stemmed from a significant increase in lung concentrations of bronchodilatory atrial natriuretic peptide and from a decrease in endothelin-1, which is associated with bronchoconstriction. A reduction in the severity of inflammation was also observed on histological examination; however, this was not accompanied by a decrease in pro-inflammatory cytokine levels or a reduction in the degree of emphysema following treatment with the GLP-1 RA [125]. On the other hand, a study of obese mice exposed to cigarette smoke extract demonstrated the efficacy of GLP-1 RA therapy in inflammation treatment, confirmed by a significant decrease in the number of CD3^+^ T lymphocytes, F4/80^+^ macrophages, and CD11b^+^ macrophages in the blood, attenuated emphysema, reduced inflammatory cell infiltration, reduced edema, and reduced microcirculation disturbances. It was suggested that GLP-1 may stimulate the regeneration of damaged pulmonary endothelium, as a reduced number of CD31+ endothelial cells undergoing apoptosis was observed in the lungs [126]. Another experimental study points to an additional positive effect of GLP-1 RAs on airway mucus homeostasis. Treatment of mice with chronic bronchitis exposed to pyocyanin by exendin-4 administration restored the expression of the forkhead box A2 (FOXA2) protein and reduced mucus production by activating the GLP-1R-PKA-PPAR-γ pathway-dependent phosphatases PTEN and PTP1B, subsequently dephosphorylating and inhibiting key kinases in the signaling pathways responsible for goblet cell hyperplasia and metaplasia [127].

The enhancement in lung function described in experimental studies corresponds to a randomized controlled trial conducted among patients with obesity and COPD, in which 40 weeks of treatment with liraglutide led to an improvement in forced vital capacity (FVC), carbon monoxide diffusion capacity (DLCO), and the COPD assessment test score [128]. No decrease in CRP, IL-6, or MCP-1 levels was observed, and the authors do not attribute the effect of liraglutide to weight loss, as this remained relatively stable throughout the study. Human observational studies have yielded some interesting results, although their number is small. A large population study involving 1252 patients with T2DM and COPD using GLP-1 RAs (dulaglutide, exenatide, liraglutide, lixisenatide, and semaglutide) showed a 30% lower risk of severe COPD exacerbations compared to the group treated with sulfonylureas [129]. Another retrospective, observational study involving 1642 patients with COPD within the US healthcare system compared disease exacerbation between patients prescribed GLP-1 RAs and other T2DM medications, DPP-4is, SGLT-2i, or sulfonylureas [130], with GLP-1 RAs being the most effective. Taking various confounding factors into account, the researchers conducted a multilevel analysis and showed that the risk of exacerbations was significantly lower among patients using GLP-1 RAs compared with those using sulfonylureas or DPP-4 inhibitors. Similar conclusions were reached in three large-scale, targeted studies involving adult patients with T2DM and active COPD enrolled in US health insurance programs, in which treatment with GLP-1 RAs was associated with a lower risk of severe or moderate COPD exacerbations compared with DPP-4 inhibitors [131]. Another retrospective cohort study involving a comparison group and 4479 patients treated with GLP-1 RAs confirmed that, after one year of therapy, these patients experienced fewer disease exacerbations, fewer prescriptions for oral corticosteroids, and a significant delay in the time to first prescription for them [132]. The key outcome measured in the studies described above was a reduction in the number of exacerbations. Nevertheless, a long-term cohort study involving 8060 matched individuals—both those using and those not using GLP-1 RAs—based on data from Taiwan’s national health insurance database showed that, in patients with T2DM and COPD, the use of GLP-1 RAs was also associated with a lower risk of all-cause mortality and cardiopulmonary complications [133]. The evidence suggesting a link between the use of GLP-1 RAs and a lower number of COPD exacerbations is promising, but requires further clinical trials and, above all, the mechanisms underlying this effect must be clarified.

## 8. GLP-1 RAs and Obstructive Sleep Apnea (OSA)

**Figure 2:**
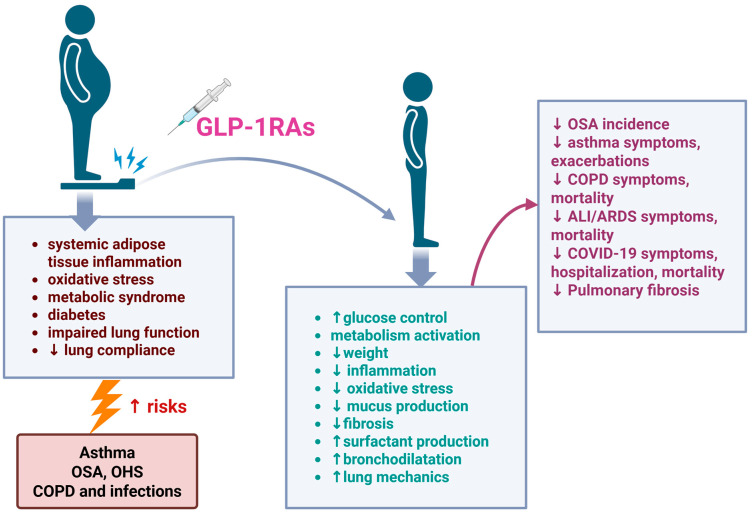
The relationship between obesity, metabolism, systemic inflammation, and respiratory diseases, as well as the role of GLP-1 receptor agonists (GLP-1 RAs). ALI/ARDS: acute lung injury/acute respiratory distress syndrome; OSA: obstructive sleep apnea; COPD: chronic obstructive pulmonary disease; OHS: obesity hypoventilation syndrome; ↓: decrease; ↑: increase.

Obstructive sleep apnea (OSA) is a sleep disorder characterized by repeated episodes of breathing cessation (apnea) or shallow breathing (hypopnea) during sleep, resulting in a decrease in blood oxygen saturation and sleep fragmentation. Apnea occurring repeatedly during each hour of sleep, resulting in intermittent hypoxia and reoxygenation, which promotes oxidative stress and systemic inflammation and has serious clinical consequences, such as cardiovascular complications, metabolic disorders, neurocognitive problems, and reduced quality of life [134,135,136]. The vast majority of people with OSA (60–70%) are overweight, which is considered the most reversible important risk factor for OSA [137,138,139]. This is associated with increased fat accumulation in the neck and upper respiratory tract, which can narrow the airways and restrict airflow. Even a small increase in body weight predicts an increase in AHI (apnea/hypopnea index) and an increased risk of moderate or severe OSA, while a 10% weight loss predicts a 26% decrease in AHI [137]. Losing excess weight reduces the accumulation of fat in the tongue, throat, and abdomen, which physically prevents external narrowing and collapse of the upper airway and throat during sleep and lowers the number of breathing arrests in OSA [140]. Not surprisingly, there has been interest in the new therapeutics used to control blood sugar in T2DM and to promote significant weight loss, namely GLP-1 RAs and dual GLP-1 RA and GIP receptor agonists, for the treatment of OSA [141,142,143,144,145]. Figure 2 illustrates the relationship between obesity, metabolism, systemic inflammation, and respiratory diseases, as well as the role of GLP-1 receptor agonists (GLP-1 RAs).

Interestingly, GLP-1 RAs show therapeutic potential in the treatment of OSA, not only by promoting weight loss through slowing gastric emptying, controlling appetite, and improving insulin sensitivity [146], but also by acting on comorbidities. Of significance is their ability to help maintain normal blood glucose levels, exert anti-inflammatory and antioxidant effects [16,23,24,147,148], and to have a beneficial effect on atherosclerosis and cardiovascular diseases [149]. It is, therefore, not surprising that GLP-1 RAs are being proposed as drugs with multifaceted effects on obesity, diabetes, and sleep apnea [150]. When discussing and summarizing the impact of GLP-1 RAs on OSA, we focused exclusively on the most relevant trials and randomized controlled trials, as several systematic reviews and meta-analyses of such studies have recently been published [151,152,153,154,155]. One of the first RCT was conducted on 359 patients with obesity and moderate or severe OSA who were reluctant or unable to use Continuous Positive Airway Pressure therapy (CPAP—the gold standard therapy for OSA), but were treated with liraglutide (3 mg) or placebo for 32 weeks, both as a supplement to diet and exercise [156]. The study showed that liraglutide caused a greater reduction in AHI compared to placebo (−12.2 vs. − 6.1 events per hour), accompanied by a greater mean percentage weight loss (−5.7% vs. −1.6%) and a greater reduction in glycated hemoglobin (HbA1c) and systolic blood pressure compared to placebo. A recent study looked at the effectiveness and side effects of liraglutide when treating patients with T2DM and severe OSA [157], who were randomly assigned to either the control group (CPAP and drug treatment without liraglutide—45 patients) or the liraglutide group (CPAP and drug treatment with liraglutide—45 patients). Three months of treatment with liraglutide, gradually increased doses from 0.6 to 1.8 mg, significantly reduced AHI from 31.0 to 26.1 events per hour, reduced mean systolic blood pressure, and increased oxygen saturation compared to the control group. No differences were detected between liraglutide and the control group in the overview of side effects. The SURMOUNT-OSA study—a phase III, multi-center RCT—was conducted in nine countries to evaluate the efficacy and safety of weekly tirzepatide (10 mg or 15 mg) applied for 52 weeks in adults with moderate-to-severe OSA and obesity [138]. A total of 469 participants were randomly assigned to receive tirzepatide or placebo in Study 1 (234 participants) or Study 2 (235 participants). In both studies, the AHI decreased significantly by up to 58.7% from baseline among participants who received tirzepatide, regardless of concomitant PAP (positive airway pressure) therapy, compared with a decrease of 3.0% from baseline among those who received placebo. Additional benefits of tirzepatide included reduced body weight, reduced hypoxia burden, reduced hsCRP concentration, and reduced systolic blood pressure. Such a significant change has important clinical implications, but it should be noted that tirzepatide is a long-acting agonist not only of the GLP-1 receptor, but also of the GIP receptor. With regard to the safety profile of tirzepatide, the most commonly reported adverse events were mild-to-moderate gastrointestinal events, occurring mainly during dose escalation. Recently, there has also been considerable interest in a triple agonist with a favorable safety profile that activates the receptors for GLP-1, GIP, and glucagon, named retatrutide. As part of the TRIUMPH RCT, an evaluation will be conducted to assess the safety and efficacy of weekly subcutaneous retatrutide in the treatment of adults with obesity and its common complication—OSA [158]. Since retatrutide, when used as monotherapy in adults with type 2 diabetes, has been shown to significantly improve glycemic control and lead to robust weight loss [159,160], it is also expected to lower the AHI.

## 9. Conclusions

A large body of evidence indicates that the use of GLP-1 RAs has a beneficial effect on the respiratory system and related disorders by acting on a plethora of symptoms. Typically used to treat obesity and diabetes, they have been shown to reduce inflammation in asthma, COPD, ALI/ARDS, and pulmonary fibrosis, and oxidative stress in OSA and COVID-19. Other favorable effects include improved lung function and a reduction in exacerbations of asthma and COPD, as well as increased survival in COVID-19, COPD, and ALI. However, the underlying causes of the drugs’ effects on lung function were not analyzed. Regrettably, some of the outcomes described are based solely on animal in vivo studies, such as the increased production of surfactant proteins in models of pulmonary fibrosis or ALI. Consequently, we still do not know whether the results obtained will apply to humans. There is also a lack of science-based evidence to determine whether GLP-1 RAs reduce the number of apneas mainly by promoting weight loss and improving metabolic health, or whether they affect respiratory control and upper airway muscle tone. In addition, there is also a paucity of studies on the long-term efficacy and safety of GLP-1 RAs for most respiratory diseases. An interesting question is whether GLP-1 RAs will be beneficial in the management of lung diseases in individuals without obesity. The main limitation of the majority of the discussed evidence is that the studied populations consist mainly of patients with diabetes and obesity. In contrast, studies conducted on non-obese animals—for example, in models of COPD or pulmonary fibrosis—indicate that GLP-1 RAs have beneficial effects. There is, therefore, a likelihood that using these treatments in lean people could also be effective; this would likely require determining appropriate doses that do not cause weight loss. In light of the above, further research is required to elucidate the mechanisms underlying the beneficial effects of GLP-1 RAs in animal models and in humans, as well as large-scale clinical trials and long-term efficacy studies to confirm their therapeutic effects. This knowledge—along with the identification of specific patient subgroups that may benefit most from GLP-1 RA therapy, the determination of optimal doses, and, ultimately, a personalized approach to patient care—may lead to the future use of GLP-1 RAs in the treatment of lung diseases.

## Figures and Tables

**Table 1:** The method and frequency of administration, and the dosage ranges for GLP-1 analogs [26,30,31].

GLP-1R Agonist	Route of Administration	Frequency of Administration	Dose
dulaglutide	subcutaneously (s.c.)	weekly	0.75–4.5 mg
liraglutide	s.c.	daily	0.6–1.8 mg
semaglutide	s.c.	weekly s.c.,	0.25–2 mg
	orally	daily orally	1.5–14 mg (tablets)
exenatide	s.c.	twice daily	5–10 μg
exenatide–LAR (long-acting-release)	s.c.	weekly	2 mg
tirzepatide	s.c.	weekly	2.5–15 mg

**Table 2:** Effects and mechanisms of GLP-1 RA treatment in AI.

GLP-1R Agonist	Study	Effects	Mechanism	References
liraglutide	in vivo	↓ lung injury ↓ number of cells in BALF ↓ cytokines in BALF and lungs ↓ mortality ↑ surfactant proteins (SP-A and SP-B) and phospholipids	↓ inflammation via TxNIP/NRPL3 inflammasome ↑ surfactant production	[38,39,40,41,42,43]
liraglutide	in vitro (ATII)	↑ surfactant protein SP-A expression in ATII cells	↓ inflammation and ↑ surfactant production via thyroid transcription factor-1 (TTF-1)	[43]
dulaglutide	in vivo	↓ lung injury ↓ number of neutrophils and macrophages in lung tissue ↓ cytokines in lungs ↓ expression of apoptotic proteins ↓ apoptosis of epithelial cells	↓ inflammation via NRPL3 and STAT3 signaling ↓ apoptosis	[44]
liraglutide	in vivo	↓ lung injury ↓ alveolar-capillary barrier dysfunction ↓ PMN extravasation	maintenance of functional endothelial barrier	[45]
	in vitro (HPMECs)	↓ endothelial barrier damage ↓ PMN migration and adhesion	↓ Rho/NF-κB signaling ↓ ICAM-1 and VCAM-1	

Note: ATII cells: alveolar epithelial type II cells; HPMECs: human pulmonary microvascular endothelial cells; ↓: decrease; ↑: increase.

## Data Availability

No new data were created or analyzed in this study. Data sharing is not applicable to this article.
